# Study of the costs of an ICU according to age groups relating to SAPS 3 gravity, stay and outcomes

**DOI:** 10.1186/cc14608

**Published:** 2015-03-16

**Authors:** MB Velasco, MA Leitão, DM Dalcomune, MB Leitão

**Affiliations:** 1 Hospital Meridional S.A., Cariacica, Brazil

## Introduction

Hospital costs are a constant concern within health, especially in the ICU. Hospital admissions and average life expectancy have been growing gradually mainly in older and critical patients. This study is aimed to observe the direct costs of patients admitted to an ICU and their relation to the SAPS 3, length of stay in the ICU and final outcome.

## Methods

A retrospective observational study in which the direct costs were studied (materials, medicines, oxygen therapy and hospital fees ) for 1,790 ICU patients from November 2013 to November 2014. The readmissions within 48 hours were excluded and also 10% of patients who had the highest and lowest costs. The remaining 1,401 patients were divided by age groups.

## Results

**Figure 1:**
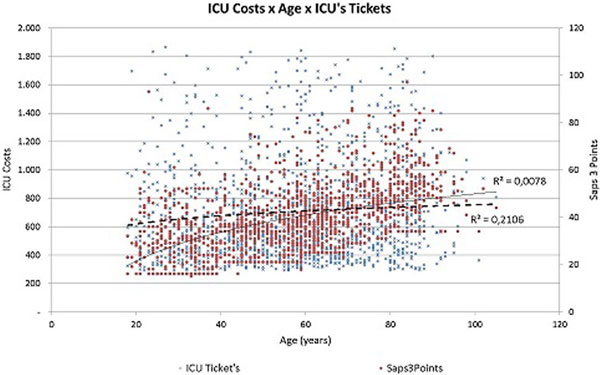


**Figure 2:**
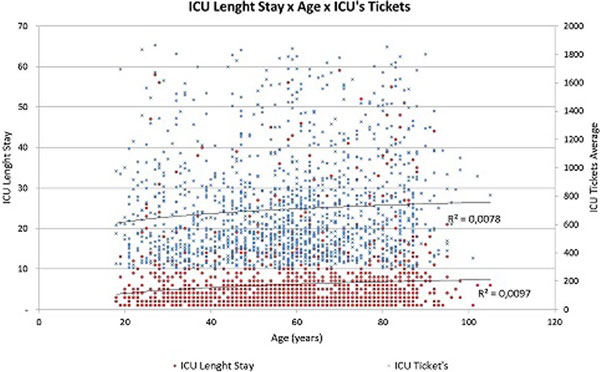


Of the patients studied, 54.6% were male. Average age was 57.8 years (18 to 105 years). The biggest ICU average cost was in the group of patients 81 to 90 years old (US$793.00), as well as longest ICU stay (9.25 days), highest SAPS 3 (53.96) and higher ICU and in-hospital mortality (14.29% and 19.25% respectively). This study shows that the direct cost of the ICU stay for older patients was higher than for younger patients. The difference was explained by the higher severity measured by SAPS 3 in the older age groups (Figure 1), and the required greater length of stay in the ICU (Figure 2). As might be expected, the mortality in the group of older patients was also significantly higher.

## Conclusion

This study showed that greater age is associated with higher severity measured by SAPS 3, higher direct costs, and higher mortality both in the ICU and in-hospital environment.

